# Kicking Back Cognitive Ageing: Leg Power Predicts Cognitive Ageing after Ten Years in Older Female Twins

**DOI:** 10.1159/000441029

**Published:** 2015-11-10

**Authors:** Claire J. Steves, Mitul M. Mehta, Stephen H.D. Jackson, Tim D. Spector

**Affiliations:** ^a^Department of Twin Research and Genetic Epidemiology, Kings College London, London, UK; ^b^Department of Clinical Gerontology, Kings College Hospital, London, UK; ^c^Centre for Neuroimaging Sciences, Institute of Psychiatry, Kings College London, London, UK

**Keywords:** Cognition, Age-related changes, Physical performance, Physical activity, Postmenopausal women, Healthy ageing, Structural MRI, Functional MRI, Twin studies, Leg power

## Abstract

**Background:**

Many observational studies have shown a protective effect of physical activity on cognitive ageing, but interventional studies have been less convincing. This may be due to short time scales of interventions, suboptimal interventional regimes or lack of lasting effect. Confounding through common genetic and developmental causes is also possible.

**Objectives:**

We aimed to test whether muscle fitness (measured by leg power) could predict cognitive change in a healthy older population over a 10-year time interval, how this performed alongside other predictors of cognitive ageing, and whether this effect was confounded by factors shared by twins. In addition, we investigated whether differences in leg power were predictive of differences in brain structure and function after 12 years of follow-up in identical twin pairs.

**Methods:**

A total of 324 healthy female twins (average age at baseline 55, range 43-73) performed the Cambridge Neuropsychological Test Automated Battery (CANTAB) at two time points 10 years apart. Linear regression modelling was used to assess the relationships between baseline leg power, physical activity and subsequent cognitive change, adjusting comprehensively for baseline covariates (including heart disease, diabetes, blood pressure, fasting blood glucose, lipids, diet, body habitus, smoking and alcohol habits, reading IQ, socioeconomic status and birthweight). A discordant twin approach was used to adjust for factors shared by twins. A subset of monozygotic pairs then underwent magnetic resonance imaging. The relationship between muscle fitness and brain structure and function was assessed using linear regression modelling and paired t tests.

**Results:**

A striking protective relationship was found between muscle fitness (leg power) and both 10-year cognitive change [fully adjusted model standardised β-coefficient (Stdβ) = 0.174, p = 0.002] and subsequent total grey matter (Stdβ = 0.362, p = 0.005). These effects were robust in discordant twin analyses, where within-pair difference in physical fitness was also predictive of within-pair difference in lateral ventricle size. There was a weak independent effect of self-reported physical activity.

**Conclusion:**

Leg power predicts both cognitive ageing and global brain structure, despite controlling for common genetics and early life environment shared by twins. Interventions targeted to improve leg power in the long term may help reach a universal goal of healthy cognitive ageing.

There is now consistent evidence from observational studies of a protective association between levels of physical activity (PA) and subsequent cognitive ageing within the healthy population [1] and subsequent risk of dementia [2]. This effect has been found across the spectrum of PA [3]. However, randomised controlled trials of PA interventions have produced inconsistent results [4,5,6], precluding firm conclusions about a causal effect. Importantly, despite a surge in recent publications showing exercise interventions lead to short-term gains in cognition, no trials have yet been long enough to test if these interventions are effective in altering the course of cognitive ageing.

More information can be gained from longitudinal observational data, although care has to be taken to avoid confounding. PA is notoriously difficult to measure: self-reported measures may reflect other factors such as optimism or knowledge of healthy lifestyles, both of which have been related to cognition in older adults; while free-living ‘objective’ measures such as accelerometry, are prone to recording bias and exhibit substantial intra-individual variability [7]. Measures of physical fitness [the capacity to perform PA (PF)] are much more tractable and include muscular fitness. They are also correlated with PA and have similar effects on health [8]. As such, measures of PF might better reflect habitual PA than either self-report or short-term objective measurement.

However, the relationship between physical fitness and cognitive ageing in non-impaired populations has not received as much attention. The Rush Memory and Aging project found a strong protective effect of muscle strength on cognitive ageing which remained after adjusting for self-reported PA [9]. A similar effect has been reported for cardiopulmonary fitness [10]. There is some evidence that lower limb fitness is particularly related to cognitive ageing: effective exercise interventions over a short time span have involved exercise of the lower limb [11]; and in a recent meta-analysis, walking speed was significantly related to subsequent change in fluid abilities [12].

Some of the association among PA, physical fitness, cognitive change and brain volumes may be explained by confounding through common genetic causes: genetically ‘fitter’ individuals may be more physically fit and more likely to engage in PA, and therefore exhibit a ‘robust phenotype’ with improved cognitive ageing trajectories and reduced atrophy due to genetic advantages. Previous twin studies have shown that up to a third of the variance in a range of physical fitness measures can be attributed to genetic effects [13]. In addition, uptake and response to exercise interventions may be driven in part by genetic [14] and developmental [15] factors which could confound the relationships observed.

Fitness may likewise reflect differences in early life factors, such as intrauterine environment, birthweight, education and socioeconomic status. There is an established literature associating early life factors and adult strength [16]. Some attenuation in the association between *cross-sectional* physical functioning and cognitive functioning has been described previously for childhood IQ and socio-economic status [17,18].

Later in life, there may also be common factors responsible for ageing in cognitive and non-cognitive systems [19]. One contender is vascular damage. The association between physical fitness and cognitive ageing may be mediated through improved cardiovascular risk factors and or diseases: PA is protective against cardiovascular disease, and both ischaemic heart disease [20] and heart failure [21] have been associated with cognitive deficits and brain atrophy in non-impaired non-stroke populations. Other generalised ageing processes, marked by telomere lengthening or the accumulation of health deficits (frailty), might also explain any relationship between physical fitness and cognitive ageing. This may be particularly important at later ages, where a complex and dynamic interplay is likely.

The key measure of physical fitness in this study is leg explosive power (LEP), which is sensitive to low-intensity PA, performing better than maximal oxygen consumption (VO_2 max_) [22]. LEP is correlated with functional ability and declines with age earlier, and more dramatically, than strength [23,24]. In a randomised exercise intervention trial, LEP was the measure most influencing functional improvement [25]. For these reasons, we considered it to be the best simple measure of physical fitness. It is measured using the Nottingham power rig, which assesses the *force* and *velocity* of leg extension. LEP is highly reliable (reliability coefficient 0.97, coefficient of variation 9.4%, over 1-week period in naïve adults), validated, and can be performed by comparatively frail older people [26].

The current study aimed to test whether greater leg power was associated with improved cognitive trajectory over a 10-year period, when adjusting for the possible effect of genetics, early development, frailty and disease which could contribute to reverse causation. Cognition was measured using a computerised battery of tests which were combined using principle components technique to assess age-related change in global cognition [27]. A twin cohort was utilised as they share genetic factors and early-life environment. Such factors are largely non-modifiable in adulthood, so if these drive the association between physical fitness and cognitive ageing, then interventions are unlikely to be effective. Conversely, if leg power is still predictive within twin pairs, it is likely that it can be modified to improve cognitive ageing. A mid-life population was chosen, thereby avoiding some of the dynamic interactions anticipated in later old age. As there is evidence that brain atrophy precedes cognitive decline in the development of cognitive impairment [28], we also tested whether within-twin differences in leg power predicted differences in pre-specified global and regional brain volumes and activation 12 years subsequently in a sample of identical twin pairs.

## Methods

### Design

This study used a longitudinal observational cohort design in a sample of 324 female twins from the TwinsUK volunteer registry. The cohort was originally set up to study ageing in women unselected for any disease, and the recruitment and participants have been described in detail elsewhere [27]. There were no selection criteria at baseline. Exclusions at follow-up were (a) death of one twin; (b) significant cerebral pathology in one twin, and (c) withdrawal from the study. Ethical approval for the study was obtained from Guys and St Thomas' Research Ethics Committee (since renamed London-Westminster).

### Variables

#### Outcome: Age-Related Cognitive Change

Subjects underwent the same seven computerised CANTAB tests [29] in 1999 and 2009, performed on a touch screen. Tests focused on cognitive domains known to be particularly sensitive to age: memory [Pattern Recognition Memory (PRM), Delayed Matching to Sample (DMS), Paired Associates Learning, Spatial Span, Spatial Working Memory] and processing speed (PRM, DMS, Simple Reaction Time and Five-Choice Reaction Time). Change in cognition was modelled using a latent difference approach, adjusting for baseline cognitive performance. Tests and methods are described further in Steves et al. [27]. Briefly, the first factor was extracted from exploratory factor analysis of seven change scores (residuals from regression of difference scores on baseline performance). This central dependent variable was denoted the age-related cognitive change (ARC) due to its significant association with age and follow-up quality of life [27].

#### Predictor 1: Muscular Fitness: LEP (1999)

Leg extension muscle power was measured in 1999 by a trained research nurse, using the Leg Extensor Power Rig (designed by Nottingham University Medical School). The seat of the power rig was adjusted so that the subject's leg was almost fully extended. The subject sat on the chair with their arms folded and their inactive leg hanging in mid-air or resting on the machine but not used as a lever. The active leg was placed on the pedal, with the heel against the lower and inner lips of the pedal, in its ‘up’ position. The flywheel was rotated by hand until a red dot appeared in the casing window and then further rotated backwards to take up the slack. The subject was then instructed to undertake a practice push, leaning slightly forward and pushing the pedal submaximally to full extension, and asked to allow it to return slowly to the start position. The whole foot remained in contact with the pedal at all times. The flywheel was then reset for the performance trials. For these, the subject was instructed to push the pedal down as fast and as hard as possible - ‘as if performing an emergency stop in a car’. Strong verbal encouragement was given by the observer. The power output was noted and then switched to zero for the next push. The pedal was returned, and the flywheel braked and rotated to the start position as before. Each subject made three attempts with 30-second rest between each. The best of three results in the dominant leg was recorded and used in this analysis.

#### Predictor 2: Self-Reported PA Questionnaire (1999)

Participants were asked: ‘During the last 12 months, how would you describe the kind of PA you performed’ at home, at work and during leisure time, using a 4-point Likert scale (inactive, light, moderate, heavy). A weighted average of the three PA level responses was calculated (see online suppl. data; for all online suppl. material, see www.karger.com/doi/10.1159/000441029). This measure was significantly related to PA measured by accelerometry in the same cohort in 2007 [30] (online suppl. table S1).

#### Covariates

Covariates measured during the baseline visit were chosen because of evidence from observational studies that these factors are also associated with cognitive ageing in non-diseased ageing populations [31]. These are listed in table 1, detailed in online supplementary materia1, and cover developmental factors, adult psychosocial variables, adult lifestyle, health measures and diseases. Additionally, analysis was adjusted for leucocyte telomere length, frailty (Rockwood Frailty index) and other physical fitness measures: grip strength, lung function and right leg lean mass (for details, see online suppl. material).

### Statistical Methods

ARC was modelled such that more positive values mean more ‘positive’ change (less decline). Missing values of predictors and covariates were imputed using multiple imputation in Stata 11 (Rubin's method). As attrition rates were low, and those lost to follow-up not significantly different from the study participants [27], no adjustment was made for loss to follow-up. Independent variables were standardised to allow comparison between the effects of the variables. An additive linear regression was performed using a hierarchical framework with five levels in order to account for distal factors before proximal ones. The levels were: developmental, adult socio-economic, lifestyle, health measures and disease. The generalised estimating equation approach was used to adjust for family relatedness, treating each participant as an individual. All models co-varied for age. No non-linear relationships or interactions significantly improved the model fit (data not shown).

For twin pair analysis, paired t tests were used to estimate the difference in outcome in discordant twin pairs defined as more than 1 SD different on LEP (adjusted for birthweight, adult height and household income, using the residuals method). This adjustment was made to counteract any effects of early life differences between the twin pairs, which might contribute to subsequent leg power, and yet not be modifiable in later life. A regression-based model estimating within-pair effects was also used (online suppl. table S2). Sensitivity analysis was performed for apolipoprotein (ApoE) ε4 and mild cognitive impairment (MCI) status (for details of assessment, see online suppl. material).

### Magnetic Resonance Imaging Sub-Study Methods

#### Participants

Twenty pairs of twins were sampled along the distribution of discordance in ARC for neuroimaging at a single time point in 2011-2012 (12 years after baseline) as a cost-effective strategy (for characteristics, see online suppl. table S3). Exclusion criteria for the magnetic resonance imaging (MRI) study were: twins discordant for psychoactive medications; twins discordant for handedness or both left handed; subsequent neurological events in year between last testing; MRI non-compatibility; presence of diabetes or ischaemic heart disease. Discordance in LEP for the MRI study was defined as >25-watt difference in baseline leg power in 1999 (25 W is a clinically meaningful difference in LEP, corresponding to approximately 10 years of difference in women of this age [32]).

#### MRI Acquisition and Processing

Subjects were acclimatised to the MRI environment in a mock-up scanner and underwent interactive training for the functional task. Scans were acquired on the twin pairs on the same day and same 1.5-tesla Signa HDx MR scanner (GE Medical Systems, Milwaukee, Wis., USA), including high-resolution T1 structural images, during a block-design choice reaction time task modelled on the choice reaction time in the CANTAB. This was the single task with the highest loading for the age-related change factor. Full details of MRI acquisition and pre-processing are supplied in the online supplementary material. In brief, structural processing was performed using Statistical Parametric Mapping (SPM8). The Diffeomorphic Anatomical Registration through Exponentiated Lie Algebra (DARTEL) technique [33] was used to create a template of all subjects' structural images for normalisation with the aim of improved registration for subsequent comparisons.

Two regions of interest - the medial temporal lobe (MTL; incorporating the hippocampus and parahippocampus) and the middle frontal gyrus - were identified using a mask from the Automated Anatomical Labeling (AAL) library. The middle frontal gyrus volume was extracted as clear anatomically delimited templates exist, and this region incorporates a large portion of the dorsolateral prefrontal cortex (DLPFC; which has no clear anatomical boundaries). Grey matter volumes for the regions of interest were extracted from the DARTEL flow output using MarsBar software. In addition, global measures of ventricular volume (sum of 3rd, 4th left and right lateral and inferior lateral ventricles), total grey matter and intracranial volume were generated using Freesurfer software (version 4.5.0; http://surfer.nmr.mgh.harvard.edu/) according to a standard pipeline. The resultant extracted volumes were used in linear regression (generalised estimating equation) and paired t tests performed in STATA11.

The functional task was a choice reaction time task with four circles around a fixation cross. Activations were contrasted in three blocks: control (fixation cross); cued (where the subject was alerted to the correct circle), and choice reaction time. Functional processing and statistical inference was performed using standard techniques in SPM8. For further details, see online supplementary material. Functional images were co-registered to that individual's MPRAGE scan (T1 image) and normalisation to standard space was achieved using the DARTEL template.

For each task, interaction contrast maps depicting differences in whole-brain activity for each twin pair were inputted into the second-level models to identify regions significantly activated in the stronger compared to the weaker twins in the task condition relative to control, and conversely, using paired t tests. Tests were made with and without adjustment for reaction time difference between the pairs measured in the scanner. Reported results were corrected for multiple comparisons based on both voxel intensity (family-wise error) and cluster extent.

## Results

In 2009, 401 subjects met the entry criteria and were invited to attend [27]. A total of 324 [127 monozygotic (MZ), 197 dizygotic (DZ)] were tested. Both study visits were conducted in the same institution, with the exception of 14 subjects tested at home in 2009 in order for physically frailer individuals to be included.

### Multivariate Analysis

Both PA [standardised β-coefficient (Stdβ) = 0.129, p = 0.028] and LEP (Stdβ = 0.184, p < 0.001) measured at baseline had independent protective effect on ARC over the subsequent 10 years (adjusting for age and developmental, psychosocial and other lifestyle factors). All models were adjusted for age, as there was an expected association between age and muscle fitness with 10 years leading to almost 0.5 SD decline in leg power (Stdβ = −0.0478, p < 0.001). The statistically strong evidence for the effect of LEP (Bonferroni adjusted p value threshold = 7.8 × 10^-3^) remained significant adjusting for health measures and disease (Stdβ = 0.174, p = 0.002, level 5 model; table 1), and including adjustment for baseline lower limb arthritis (data not shown).

LEP had the most consistent and largest effect size of all covariates, but the absolute effect size was modest: an increase in LEP of 40 W produced an 18% SD improvement in ARC (equivalent to 3.3 years difference in age); an increase from light activity to moderate activity for 8 h a day led to a 13% SD improvement in ARC. Of other covariates, it is notable that fasting glucose levels, adjusted for diabetic status, were positively related to ARC (higher fasting blood glucose in the normal range was protective).

### Additional Analysis

Point estimates and significances remained unchanged, excluding 9 individuals characterised as having amnestic MCI (2.8% of the cohort). ApoE data were available on only 155 individuals in the sample. Of these, 10 individuals were homozygous for ApoE ε4. ApoE ε4 homozygous status was itself strongly associated with ARC (β −0.54, p = 0.002). Sensitivity analysis excluding these individuals showed no substantive changes in point estimates or significances of any independent variable (data not shown).

As the cognitive battery was performed on a computer, to check whether computer usage over the follow-up interval could have biased results, models were also adjusted for a proxy for computer usage (possessing an email address), with little change in results (table 2; F = 0.35, p = 0.557). The finding for LEP was robust to inclusion of other physical measures (online suppl. table S4). Neither grip strength (Stdβ = −0.031, p = 0.588), lung function (Stdβ = 0.060, p = 0.226), nor right leg lean mass (Stdβ = 0.034, p = 0.546) were predictive of ARC when exchanged for LEP. Neither frailty (frailty index) nor telomere length attenuated the relationship between LEP and ARC (table 2).

### Discordant Twin Analyses

**Fig. 1:**
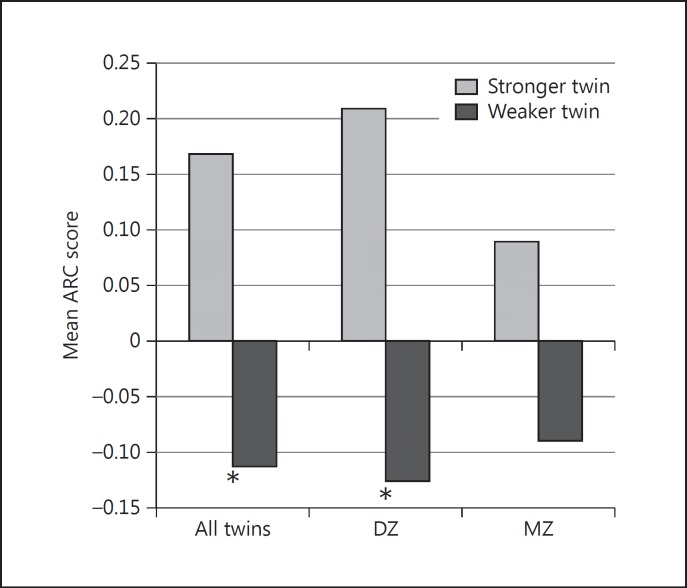
Age-related change in twins discordant for LEP. LEP discordance was defined as a difference of >1 SD. There were 28 DZ and 14 MZ twins. * p < 0.05, paired t test, one tailed.

For this analysis, complete case data were used (n = 221, 118 pairs), yielding 42 pairs discordant for LEP. On average, twins who were stronger at baseline had significantly less deterioration in cognition than their weaker sisters. The same significant effect was apparent in the DZ twins alone, but did not reach significance in MZ twins (numbers in the MZ group were small; table 3; fig. 1). Similar analysis for PA showed better average ARC scores in twins reporting more PA (adjusted for birthweight, height and household income), but the effects were not significant in any group (more active/less active ARC scores, respectively: all twins 0.15/0.02; DZ 0.09/-0.04; MZ 0.26/0.10). Alternative regression modelling, using a within-twin and between-family difference approach in the full sample, showed similar results (online suppl. table S2).

### Imaging Results

#### Structural Results

Participants in the imaging subset were representative of the whole sample (online suppl. table S3). In all individuals, ARC was associated with larger MTL volumes and smaller ventricles (table 4). LEP had a positive association with total grey matter volume 12 years later (age-only adjusted model Stdβ = 0.362, p = 0.005). This effect was also seen in models adjusting for height and birthweight, shown in table 5. No association was found between LEP and ventricle size or the regions of interest, the MTL and middle frontal gyrus (data for regions not shown).

#### Discordant Twins Analysis

There were 9 twin pairs more than 25 W discordant in LEP (adjusted for birthweight). The weaker and stronger twins were not significantly different with regard to height (p = 0.37) or intracranial volume (p = 0.77).

Stronger twins had significantly more grey matter, but no regional differences in grey matter were found in the regions of interest. Ventricular volume was markedly different, with weaker twins having 26% larger ventricles than their stronger sisters (paired t test, one-sided p = 0.025; table 6).

#### Brain Structure, LEP and Cognitive Change

The direction of effect of LEP on ARC within this smaller sample (n = 41) was the same as in the larger study (Stdβ = 0.159, p = 0.157). The association of grey matter with ARC was significant adjusting for age alone (Stdβ = 0.195, p = 0.024). This effect was significantly attenuated when adjusting additionally for LEP (Stdβ = 0.067, p = 0.621).

#### Functional Imaging Results

**Fig. 2:**
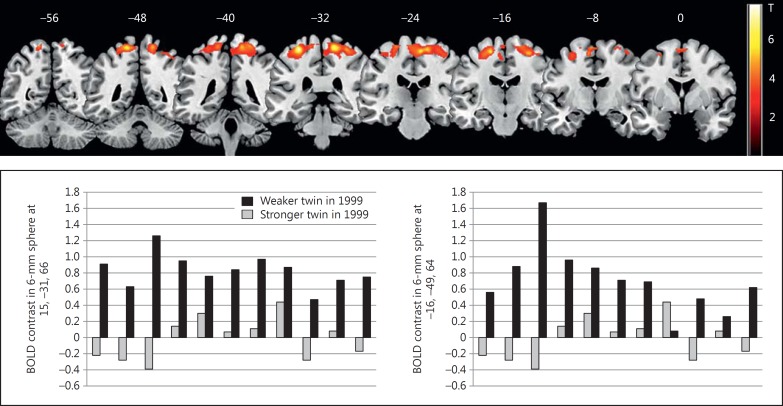
Brain activation in 2011 during a cued reaction time task in stronger compared to weaker twins in 1999. Upper panel: Significantly increased brain activity in stronger twins (1999) during cued reaction times compared to the control condition. Coronal slices (posterior to anterior) are shown overlaid with the difference in BOLD signal coloured in red/yellow. Lower panel: Bar charts showing the BOLD contrast values per twin pair in the CuedRT relative to the control condition around the peak (6-mm sphere) in the right primary motor cortex peak (left) and the left superior parietal cortex (right).

Task-specific regions identified in a previous study population were the left motor cortices, left supplementary motor area and bilateral cerebellum [34]. Similar maps were produced in the current study (available on request). For the cued task, stronger twins showed significantly more activation in the primary motor cortex bilaterally and the somatosensory association cortex on the left (BA 5). This effect was robust to adjustment for in-scanner performance (table 7; fig. 2). In the choice reaction task, there were no significant differences between stronger and weaker twins, although it is notable that, for the sub-threshold, the stronger twins had higher activation values in the right primary motor cortex (table 7). Extracted contrast data for each twin at the peak of the right primary motor cortex cluster confirmed that stronger twins positively activated this region (fig. 2). There were no regions showing greater activation in the weaker relative to the stronger twins in either task.

## Discussion

In this study of non-demented older female volunteer twins, we found consistent and strong evidence that *increased* leg power at baseline was associated with *improved* cognitive ageing over the following 10 years. In addition, in a discordant twin study, increased leg power within twin pairs was associated with greater grey matter volumes and greater task-related BOLD activation after 12 years. Within the MRI sub-study, global grey volume was significantly related to ARC. Adjustment for baseline leg power significantly reduced the relationship between grey matter and cognitive change, suggesting that prior physical power and global grey matter volume may be on the same causal pathway affecting cognitive ageing.

Twin modelling showed that these effects were not explained by confounding through genotype or shared environment. Likewise, a comprehensive study co-varying for individual-specific developmental, lifestyle and health measures indicates that reverse causation is not likely, and the effect is not likely to be mediated through frailty, generalised ageing as marked by telomere length, or disease. To check that this effect was not restricted to amnestic MCI cases and ApoE ε4 homozygotes, we performed sensitivity analyses which did not change the results.

The relationship between LEP and ARC was not attenuated by adding other fitness measures, such as forced expiratory volume (FEV_1_) or grip strength, and neither of these measures was associated with ARC when LEP was excluded from the model. There was some evidence of a small independent effect of self-reported PA. Both LEP (1999) and the self-reported PA measure were significantly related to measures using accelerometry performed in a separate sample of the TwinsUK cohort (online suppl. table S1), as has been reported in other studies [35].

To our knowledge, this is the first study to show an apparent effect of lower limb power on cognitive change in a normal population. Given its importance, confirmatory longitudinal studies are needed. FEV_1_, a composite measure of respiratory muscle *power* and bronchial resistance, has a reported effect on cognitive performance and cognitive ageing [36]. In the present study, LEP (power rig) and lung function (FEV_1_) were correlated (r = 0.38, p > 0.001). In our study and others, adjustment for FEV_1_ did not affect the relationship observed between leg power and cognitive change [9]. Therefore, it is more likely that the observed effect is a consequence of measuring lower limb function, rather than muscle *power* specifically.

With regard to the neuroimaging results, the hypothesis that PA spares brain tissue has support from a series of studies conducted almost exclusively by the same group [37], so this study forms an important replication. A few cross-sectional studies from other groups, have also replicated an association between PA and whole grey volumes [38]. The results of the present imaging sub-study, while limited by study numbers, are novel because of the control garnered by use of identical twins, and for two additional important reasons. First, most imaging studies to date have assessed physical fitness using direct or proxy measures of aerobic fitness or VO_2 max_. This is the first study linking a power of large leg muscular response to brain changes. Future studies are needed to unpick whether aerobic measures, leg power measures or other measures of fitness are independently related to brain changes, or whether they are related through a common mechanism.

Second, few studies have followed fitness effects on cognition and brain structure or function over more than 10 years. Published interventional studies are limited to <1 year (although some have longer follow-ups). Moreover, the inter-individual differences in this study were generated in ‘real life’ as opposed to trial interventions, which may have limited bearing on what is possible in the long term.

The limitation of the imaging sub-study design is that it cannot prove causality despite the longitudinal nature of the study. It is possible that brain functional differences in motor response, for example salience of motor experiences, led to increased LEP at baseline and the follow-up MRI reflects stability in this phenomenon. However, we interpret our findings within the context of the 7 interventional studies showing fMRI changes with short-term interventions, supporting the inference that muscular fitness leads to increased grey volumes and task-related functional activity [39].

This study therefore adds to a body of literature indicating the importance of physical fitness in cognitive and brain ageing, and is novel in focusing on muscular fitness. Strength, replication, lack of evidence for confounding and dose response (linear association) all support the probability of a causal relationship.

Despite a recent surge in publications, the mechanisms behind this association are not yet clear. Four potential mechanisms have been identified: cardiovascular, immunological, neuroendocrine and neurotrophic signalling. This study lends no support for cardiovascular factors mediating the relationship, although small vessel changes cannot be excluded. Age-related changes in immune function and inflammation are significantly reduced in individuals with a life-long history of high-level exercise [40]. Neurotrophic factors such as vascular endothelial factor, brain-derived neurotrophic factor, insulin-like growth factor 1 and fibroblast growth factor 2 may play a role in promoting angiogenesis, synaptogenesis and neurogenesis. We aim to follow up this study with a physical exercise intervention in twins discordant for physical fitness to investigate whether cognitive function of the twins converges, or whether the stronger twins maintain their advantage - and the extent to which this is explained by changes in immune and neurotrophic markers.

### Strengths and Limitations

The longitudinal follow-up over 10 years in this study is a substantial strength because effects may take time to become apparent. In particular, learning effects were diminished by the long interval. Additionally, the wealth of data at baseline and collateral data from the larger TwinsUK cohort are significant strengths.

However, as with all studies, there are some limitations. First, this cohort represents relatively educated women of middling occupational status who have marginally better health status than the general population [27]. The age domain is between 43 and 73 at first testing, following women in midlife to early old age, and therefore does not inform on changes occurring in older ages. Further studies are required to ascertain whether the findings can be generalised to men.

Indeed, LEP may have particular relevance as a fitness measure in older women who are relatively less active or less likely to engage in leisure time PA. Low-intensity general exercise, such as progressive walking, was seen to increase muscle power significantly in just 12 weeks, while neither aerobic capacity (VO_2 max_) nor quadriceps muscle cross-sectional area showed significant change [22]. Unfortunately, the study did not assess aerobic measures of physical fitness (e.g. VO_2 max_) for comparison with LEP. However, there is a strong relationship between oxygen uptake and leg power output up to VO_2 max_ which others have suggested indicates that leg power also represents a clinical measure of aerobic exercise performance [41], as well as its relevance for daily function and independence. Unfortunately, due to logistic and funding constraints, follow-up measures were not performed.

The study cognitive battery was focused on tasks measuring processing speed and visual memory and did not capture change in language performance or verbal memory. Testing was only performed at two time points, limiting the analysis to linear change. For the discordant twins analysis and the MRI sub-study, the size of the study was a limitation, and assessment of the direction of association was limited by the fact that MRI measures were not available at the start of the study. As with all studies, replication - in this case in another twin cohort - would be advisable.

## Conclusion

This study found that greater muscular fitness - as measured by leg power - is associated with improved cognitive ageing over the subsequent 10 years in non-impaired community living women. In this study population, this effect was greater than, and independent of, the effect of other lifestyle and health factors reported in the literature and tested here. LEP in women was associated with objective PA measures. There was weak evidence of an independent effect of self-reported PA on cognitive ageing. The effect of LEP on both cognition and whole-brain volumes was not confounded by factors shared by twins, i.e. age, sex, genotype, in utero factors and family background, and as such provides the first study of its kind.

This study lends no evidence for common causation of muscular fitness and cognitive ageing by genetic factors, shared environmental factors, premorbid intelligence, socioeconomic factors, frailty or cell ageing (via telomeres). Likewise, the specificity of leg fitness is evidence against a circulating common cause. This leaves two remaining explanations: LEP and cognitive ageing may be associated due to a shared mechanism which is independent of genetic and many developmental factors and specific to lower limb and/or speed and coordination of muscle function, and which affects lower limb power before cognition. If so, this study would indicate that a search for common factors underpinning brain and neuromuscular ageing should begin with non-genetic mechanisms - for example cellular changes in response to the environment found in both the brain and the neuromuscular unit.

The alternative hypothesis is that leg power is a sensitive marker of the kind of PA that can influence cognitive ageing. The principle of parsimony would favour this latter explanation. If so, interventional trials aimed at improving leg power over the long term may be fruitful in the search for strategies to improve cognitive ageing in the healthy population.

## Supplementary Material

Supplementary Data

Supplementary Tables

## Figures and Tables

**Table 1:** Multiple linear regression of factors predicting 10-year age-related change in cognition

IDs	Level 5: disease		Level 4: health measures		Level 3: lifestyle		Level 2: adult psychosocial		Level 1: developmental	
	β	p	β	p	β	p	^β^	p	^β^	p
Age (per year)	**–0.052**	**<0.001**	**–0.054**	**<0.001**	**0.058**	**<0.001**	**–0.064**	**<0.001**	**–0.067**	**<0.001**
Birthweight	0.011	n.s.	−0.007	n.s.	−0.006	n.s.	0.010	n.s.	0.014	n.s.
Adult height	0.038	n.s.	0.031	n.s.	0.020	n.s.	0.069	n.s.	0.077	n.s.
Adult ability	0.117	0.069	0.110	0.050	0.113	0.082	0.085	n.s.	0.090	n.s.
Occupation	0.043	n.s.	0.054	n.s.	0.038	n.s.	0.049	n.s.		
Household income	0.063	n.s.	0.057	n.s.	0.062	n.s.	0.092	n.s.		
Mental health score	0.030	n.s.	0.030	n.s.	0.018	n.s.	0.003	n.s.		
Smoking	−0.034	n.s.	−0.039	n.s.	−0.025	n.s.				
Alcohol	−0.045	n.s.	−0.015	n.s.	−0.021	n.s.				
Dietary saturated fat	−0.144	0.017	−0.119	0.045	−0.105	0.075				

Total vegetables	−0.065	n.s.	−0.058	n.s.	−0.063	n.s.				
Leg extensor power	**0.174**	**0.002**	**0.188**	**0.001**	**0.184**	**0.001**				
Physical activity	0.107	0.056	0.122	0.033	0.129	0.028				
Blood pressure	−0.125	0.017	−0.120	0.023						
Waist-hip ratio	−0.038	n.s.	−0.036	n.s.						
Glucose	***–0.210***	***<0.008***	0.012	n.s.						
TChol/HDL ratio	0.062	n.s.	0.061	n.s.						
Ischaemic heart disease	−0.300	n.s.								
Diabetes (T2)	**–1.93**	**0.002**								

Age-related change was modelled using the ARC factor (first factor of change in 7 computerised neuropsychometric tests from the CANTAB battery, over 10 years). Results are presented using a hierarchical framework of levels ranging from developmental effects to adult disease. Missing values were imputed using multiple imputation. Betas are all standardised, except in the case of diseases, occupation and age. Occupation is modelled as a dummy variable with manual = 1, non-manual = 0. Text in bold indicates factors significant with Bonferroni correction. p values are provided for trends (two-tailed significance between 0.1 and 0.00778). Where findings were in the opposite direction to expected, they are in italics.

**Table 2:** Reduced models for predictors of age related change in cognition

1999 IDs	Model 1		Model 2 (adjusting for email status in 2011)		Model 3 (adjusting for frailty index in 2007)		Model 4 (adjusting for telomere length ~2000)	
	β	p	^β^	p	^β^	p	^β^	p
Age	**–0.058**	**<0.001**	**–0.057**	**<0.001**	**–0.054**	**<0.001**	**–0.058**	**<0.001**
Adult ability	0.110	0.074	0.102	0.108	0.103	0.087	0.101	0.114
Percent dietary saturated fat	−0.104	0.063	−0.102	0.068	−0.099	0.075	−0.103	0.083
Leg extensor power	**0.191**	**<0.001**	**0.186**	**0.001**	**0.178**	**0.001**	**0.180**	**0.002**
Physical activity	0.109	0.043	0.109	0.045	0.098	0.071	0.107	0.047
Systolic blood pressure	−0.124	0.015	−0.121	0.015	−0.102	0.034	−0.114	0.025
Glucose level	*–0.190*	*<0.016*	*–0.192*	*<0.015*	*–0.193*	*<0.016*	*–0.174*	*<0.024*
Diabetes status	**–1.81**	**0.002**	**–1.81**	**0.002**	**–1.77**	**0.003**	**–1.69**	**0.003**
Email status (2011)	–	–	0.069	0.557	–	–	–	–
Frailty index (2007)	–	–	–	–	−0.104	0.098	–	–
WC telomere length (qPCR)							0.053	0.502

Age-related change was modelled using the ARC factor (first factor of change in 7 computerised neuropsychometric tests from the CANTAB battery, over 10 years). Reduced models contain only factors reaching a nominal p < 0.10). Missing values were imputed using multiple imputation. The ßs are all standardised, except in the cases of age and diabetes (dummy variable diabetic = 1, non-diabetic = 0). Text in bold indicates factors significant with Bonferroni correction. Where findings were in the opposite direction to expected, they are in italics. WC = White cell.

**Table 3:** Paired t tests of ARC mean in LEP discordant pairs

Discordant pairs	Mean ARC		Difference, one-tailed p
	stronger twin	weaker twin	
All twins (n = 42)	0.169	−0.112	0.022
DZ (n = 28)	0.209	−0.125	0.040
MZ (n = 14)	0.089	−0.089	0.174

ARC = Age related-change in CANTAB performance over 10 years; LEP = LEP at baseline adjusted for birthweight, height and household income.

**Table 4:** Relationship between brain structures and age-related change

Structures	Model 1		Model 2		Model 3	
	β	p	β	p	β	p
Total grey matter/ICV	0.139	0.055	0.195	0.024	0.249	0.137
Ventricles/ICV	−0.366	<0.001	−0.164	0.082	−0.384	0.004
Left MTL^a^	0.287	<0.001	0.277	0.005	0.376	0.013
Right MTL^a^	0.219	0.005	0.112	0.335	0.228	0.145

Rows represent separate models. ICV = Intracranial volume. Model 1–adjusted for age, adult ability, % saturated fat, LEP, physical activity and systolic blood pressure. Model 2–adjusted for age alone. Model 3–no adjustment.

a Regional grey matter measures are adjusted for intracranial volume and brain folding during processing.

**Table 5:** Relationship between LEP in 1999 and brain volumes in 2011

Dependent variables	Model 1		Model 2		Model 3	
	β for LEP	p	β for LEP	p	β for LEP	p
Ventricles/ICV	−0.030	0.853	−0.167	0.224	−0.161	0.209
Total grey/ICV	0.435	0.009	0.427	0.002	0.362	0.005

Standardised ßs are presented. Model 1–adjusting for birthweight, height and age. Model 2–adjusting for height and age. Model 3 - adjusting for age alone.

**Table 6:** Total and regional brain volumes in 2011 in identical twin pairs discordant for LEP in 1999 (adjusted for birthweight)

Structure	Mean weaker twin (LEP in 1999)	Mean stronger twin (LEP in 1999)	Difference	Paired t test, one-tailed p
Ventricles/ICV	0.523	−0.016	0.540	0.025
Total grey matter/ICV	−0.523	0.053	−0.577	0.025
Left MTL	0.257	−0.003	0.261	0.890
Right MTL	0.153	−0.175	0.329	0.843
Left middle frontal gyrus	0.149	0.185	−0.036	0.444
Right middle frontal gyrus	0.335	0.312	0.023	0.550

ICV = Intracranial volume. Volumes are expressed as standardized scores for the whole sample. A negative difference score indicates the weaker twins have on average smaller volumes; conversely, positive difference scores mean the weaker twins have larger volumes.

**Table 7:** Clusters activating in the reaction time tasks

Task Anatomical region	BA	FWE-corr voxel-wise p	Cluster size (voxels), k_E_	FWE-corr peak p	Uncorrected peak p	MNI coordinates		
						x	y	z
Choice RT task (unadjusted)								
R precentral	6	0.335	1,410	0.735	5.20e–05	28	−18	58
R precentral	6			0.979	2.79e–04	20	−19	58
R precuneus	4/5			0.911	3.91e–04	10	−40	64

Choice RT task^a^								
R precentral	6	0.356	1,380	0.875	9.10e–05	28	−18	58
R precuneus	4/5			0.997	4.90e–04	9	−40	66
R precentral	6			0.997	5.16e–04	20	−19	58

Cued RT task (unadjusted)								
R precentral	4	3.76e–05	9,551	0.459	2.06e–05	15	−31	66
L parietal superior	5			0.530	2.82e–05	−16	−49	66
R paracentral lobule	4			0.767	7.78e–05	8	−24	61

Cued RT task^a^								
L precentral	4	5.44e–05	8,754	0.455	1.52e–05	−30	−31	64
R precentral	4			0.731	5.17e–05	15	−31	66
L parietal superior	5			0.767	6.12e–05	−16	−49	66

Clusters listed where using a peak threshold of p < 0.01, cluster level p < 0.05 (uncorrected) and minimum extent 10 voxels. RT = Reaction time; BA = Brodmann area; FWE = family-wise error; MNI = Montreal Neurological Institute.

a Adjusting for scanner cued RT.
