# Supplementary material for: Kicking Back Cognitive Ageing: Leg Power Predicts Cognitive Ageing after Ten Years in Older Female Twins
Source: Gerontology. 2015 Nov 10;62(2):138–49. doi: 10.1159/000441029 (PMC4789972; doi:10.1159/000441029)
Supplement: Supplementary file 1 — Supplementary Data [file ger-0062-0138-s01.docx]

**Supplementary Methods**

**Physical Activity score generation**

A weighted average physical activity level was calculated according to the following formulae:

Working: Total PA = LeisurePA*1 + workPA*5 + homePA*8

Retired / not employed: Total PA = LeisurePA*1 + homePA*13

**Leg Power Measurement**

**Baseline covariates (1999)**

Blood for glucose and lipids in 1999 were extracted in the seated/semi-recumbent position after overnight fasting (>8 hours). Glucose was assayed on a Johnson and Johnson Vitros Ektachem machine, Cholesterol/HDL were assayed, using the colorimetric Enzymatic method (Roche), by the Dept of Chemical Pathology, St Thomas’ Hospital. At the same 1999 visit, reported birth weight, smoking history (pack years), occupation, household income, history of ischaemic heart disease and diabetes, were entered directly onto an electronic record. Three dietary covariates, assessed using a pre-completed validated Food Frequency Questionnaire, [[115](#_ENREF_115)] were: Dietary saturated fat content/total fat consumption (adjusted for total energy consumption), reported vegetable portion consumption/wk and alcohol (converted to g/day using a program assigned alcohol/portion size*frequency [[116](#_ENREF_116)]). Affective status was likewise assessed using the total score of the GHQ 12 [[117](#_ENREF_117)]. Adult reading IQ and literacy were assessed using the NART [[118](#_ENREF_118)] [[119](#_ENREF_119)] [[120](#_ENREF_120)]. The subject was instructed to read down the list of words presented on a word card. All responses were reinforced with an encouraging statement.

Next, during the 1999 visit, systolic blood pressure was measured by nursing staff with the subject in the seated position after 3 minutes rest using the Omron Automatic Digital Blood Pressure Monitor Model HEM-713C. Three measurements were taken with the arm supported with the correct cuff at the level of the heart, with at least one minute rest between estimations. An average of the second and third measures was used. Height and weight were measured without footwear using a stadometer and calibrated scales. Waist and hip measurements were taken, with the subjects wearing underclothes, and standing with arms by their sides, tape resting on the skin.

**Additional covariates**

APOE status was later assessed using DNA extracted from spun separated EDTA samples, using Nucleon BACC3 Genomic DNA Extraction Kits (GE Healthcare, Buckinghamshire) and frozen at -80C and later assayed using TaqMan SNP genotyping performed on an ABI Prism 7900HT and analyzed using SDS software, according to the manufacturer’s instructions (Applied Biosystems, Warrington, UK). APOE data for subjects with ambiguous genotypes were treated as missing. White cell telomere length was assessed on leukocytes by PCR on average 8.7 (median 9.2) years prior to the second cognitive testing (for details see [[121](#_ENREF_121)]).

A Rockwood Frailty Index (FI), derived from the number, rather than the nature of health problems, was created from the Healthy Ageing Twin Study data from 2007-10, in which this study is nested [[45](#_ENREF_45)], employing a well-defined method to create a proportion of deficits [[122](#_ENREF_122)]. This measure of frailty is comparable to the Fried phenotype, (Rockwood, Andrew et al.), is related to age, and predicts adverse health states[[123](#_ENREF_123)]. 39 domains were constructed from questionnaire data and clinical tests. Due to the expected γ distribution, the square root of the resulting FI was incorporated into models [[124](#_ENREF_124)].

At the 1999 visit, lean mass was assessed using body composition, measured by the Hologic QDR 2000 Plus by two research nurses trained to carry out this procedure. Similarly, the Vitalograph spirometer was used to assess lung function after a period of rest. All manoeuvres were performed with the subject standing according to standard protocols. Grip strength was measured using a hand held dynamometer during separate twin visits between 2002-2005.

MCI Status: Petersen’s criteria were used to detect likely MCI in 2009 [[125](#_ENREF_125)]. Subjective memory complaint was defined by the CAMDEX questionnaire [[126](#_ENREF_126)], objective memory impairment as having a CANTAB paired associates learning (PAL) total error score of 1.5sd worse than average (>47 errors). Criteria of preserved general cognitive function and near normal functioning, applied to all participants.

#### MRI acquisition

For the structural scanning, a high-resolution T1-weighted magnetization-prepared rapid gradient echo (MP-RAGE) 3D MRI sequence with 180 contiguous sagittal slices was performed (repetition time = 8.6 ms; echo time = 3.8 ms; inversion time = 1000 ms; field of view = 240 mm × 240 mm; slice thickness = 1.2 mm; slice gap 0 mm; flip angle = 8**°** ; matrix = 192 × 192). For the functional imaging 41 near axial slices of gradient echo echoplanar (EPI) imaging were acquired over 110 time points (repetition time = 3000 ms; echo time = 40 ms; field of view = 211 mm × 211 mm; slice thickness = 0.3 mm; slice gap 3.3 mm; flip angle = 90**°** ; matrix = 64 × 64).

**Functional task: choice reaction time.** A block-design choice reaction time task was then performed, modeled on the choice reaction time in the CANTAB. Control, cued and choice reaction time task blocks were presented on a screen in the scanner, identical to the simulation scanner. Subjects held a joystick in their right hand, as in the simulated task. The control task consisted of a fixation cross with six trials each lasting three seconds. The choice task followed, with six trials each lasting five seconds. Four black circles appeared on a white background in North, South, East and West positions. In each trial, after a variable delay to reduce the effect of anticipation [[127](#_ENREF_127)], a yellow spot would appear in one of the circles. The subject was instructed to move the joystick in the direction of the circle highlighted as soon as possible. After six trials the cued block began. Again four circles would appear. One of the circles was highlighted with a slightly thicker black outline, and the subject was trained that this would be where the yellow signal would come after a variable delay, and to wait for the yellow signal before responding by moving the joystick in that direction. Again six trials, each lasting 5 seconds were completed. This cycle of Control, ChoiceRT and Cued blocks was repeated 4 times, and the task ended with a final control block. In total, the task took 330 seconds. No instruction or break was made during the task.

**Pre-processing**

Structural processing was performed using Statistical Parametric Mapping (SPM8) software (http://www.fil.ion.ucl.ac.uk/spm/software/ spm8). The structural MR images were converted to axial slices and origins reset to the anterior commissure. Images were segmented into grey matter (GM), white matter and cerebrospinal fluid using the standard unified segmentation model in SPM8 [[128](#_ENREF_128)]. Then, GM population templates were generated from the entire image dataset using the Diffeomorphic Anatomical Registration through Exponentiated Lie Algebra (DARTEL) technique [[54](#_ENREF_54)]. This method produces a template based on this population of older female brains, leading to improved registration. After an initial affine registration of the GM DARTEL templates to the tissue probability maps in Montreal Neurological Institute (MNI) space (http://www.mni.mcgill.ca/), non-linear warping of GM images was performed to the DARTEL GM template in MNI space with a 1.5mm cubic resolution. The GMV at each voxel was obtained through modulation. The GMV images were smoothed with a Gaussian kernel with a FWHM of 8mm.

**Functional pre-processing.** In SPM 8, origins of the functional images were reset to the anterior commissure. Slice timing correction and realignment of the images were performed for each subject. Functional images were co-registered to that individual’s MPRAGE scan (T1 image). Normalisation to standard space was achieved using the DARTEL template from the structural pre-processing (above), the flow field for that individual and the coregistered, resliced images.

**Functional Model design**

First level modelling analysed twin pairs together in order of LEP performance. The design matrix incorporated block timings, estimated delay of the BOLD signal (convolution of blocks with the canonical haemodynamic response function), six movement parameters (translations and rotations) for the individual during the time series. Any errors in response made by the participants were added to the model as events along the time series. A high pass filter removed low frequency signals (>128s), and AR(1) was used to correct for first-order autocorrelation. Restricted maximum likelihood method was used for parameter estimation.

For each task, cued and choice reaction time, interaction contrast maps depicting differences in whole brain activity for each twin pair were inputted into the second level models and compared using paired t-tests. This enabled identification of regions activated significantly more in the stronger compared to the weaker twins in the task condition relative to control, and conversely. Models were also adjusted for reaction time difference between the pairs measured in the scanner, entered as a covariate. The threshold for peak level height used was p<0.01(uncorrected) with a minimum voxel extent of 10. Results were corrected for multiple comparisons based on cluster extent. Voxel intensities also significant after multiple comparisons correction (family-wise error) are also reported.
