# Supplementary material for: Kicking Back Cognitive Ageing: Leg Power Predicts Cognitive Ageing after Ten Years in Older Female Twins
Source: Gerontology. 2015 Nov 10;62(2):138–49. doi: 10.1159/000441029 (PMC4789972; doi:10.1159/000441029)
Supplement: Supplementary file 2 — Supplementary Tables [file ger-0062-0138-s02.docx]

**Supplementary Tables**

Table S1 Multivariate regression of LEP and self-reported physical activity in 1999 on accelerometry measures in 2009 in TwinsUK cohort

| **1999 variables**  **n=671** | **Energy Expenditure in accelerometry 2007** | | **Sedentary time in accelerometry 2007** | |
| --- | --- | --- | --- | --- |
|  | Beta | p value | Beta | p value |
| LEP 1999 | 0.074 | 0.051 | -0.077 | 0.043 |
| Physical Activity 1999 | 0.084 | 0.013 | -0.109 | 0.002 |

Models adjusting for age.

Table S2 The relationship between LEP and age related change in cognition within and between twin pairs.

|  | **All**  **112 pairs** | | **DZs**  **n=74 pairs** | | **MZs**  **n=38 pairs** | |
| --- | --- | --- | --- | --- | --- | --- |
|  | beta | p value | beta | p value | beta | p value |
| **LEP between pairs** | 0.075 | 0.291 | 0.103 | 0.316 | -0.094 | 0.358 |
| **LEP within pairs** | 0.272 | 0.006 | 0.301 | 0.006 | 0.194 | 0.361 |

LEP, Leg Explosive Power. Models adjusted for age and IQ/education and twin-pair differences in ‘fixed’ factors correlated with LEP, birthweight, height and household income. The within pair effect is free of confounding of factors shared by twins (This method is detailed in Carlin, J.B., et al., *Regression models for twin studies: a critical review.* Int J Epidemiol, 2005. **34**(5): p. 1089-99.)

Table S3 Participant characteristics

| **Characteristic** | **Whole sample** (n=324) | **Imaging subset** (n=41) |
| --- | --- | --- |
| Age,  mean (sd) | 66.1 (7.3) | 64.0 (7.2) |
| MMSE,  mean (sd) | 29.0 (1.15) | 29.2 (0.83) |
| SBP,  mean (sd) | 120 (15) | 117 (16) |
| LEP,  mean (sd) | 102 (40) | 110 (40) |
| % saturated fat,  mean (sd) | 36 (5.5) | 37 (5.2) |
| Physical activity,  mean (sd) | 2.72 (0.53) | 2.70 (0.54) |
| NART,  mean (sd) | 115 (9.5) | 117 (7.9) |
| ARC,  mean (sd) | 0 (1) | 0.07 (1.04) |

MMSE Mini-Mental State Examination, SBP Systolic Blood Pressure, LEP Leg Explosive Power, % saturated fat= (saturated fat intake/total fat intake)*100, NART National Adult Reading Test predicted verbal IQ, ARC Age-related Change Factor.

**Table S4: Additional physical measures**

| **1999 IDs** | **Reduced model with FEV1** | | **Reduced model with grip strength** | | **Reduced model + lean leg mass (right)** | |
| --- | --- | --- | --- | --- | --- | --- |
|  | Beta | p value | Beta | p value | Beta | p value |
| Age | -0.058 | <0.001 | -0.061 | <0.001 | -0.058 | <0.001 |
| Adult ability | 0.110 | 0.075 | 0.111 | 0.070 | 0.109 | 0.076 |
| Percent saturated fat | -0.105 | 0.062 | -0.103 | 0.069 | -0.106 | 0.060 |
| Leg Extensor power | 0.192 | 0.001 | 0.202 | <0.001 | 0.187 | 0.001 |
| Physical Activity | 0.109 | 0.043 | 0.115 | 0.037 | 0.108 | 0.046 |
| Systolic BP | -0.124 | 0.015 | -0.120 | 0.020 | -0.126 | 0.013 |
| Glucose level | 0.190 | 0.016 | 0.187 | 0.018 | 0.188 | 0.017 |
| Diabetes status | -1.81 | 0.002 | -1.79 | 0.002 | -1.82 | 0.002 |
| FEV1 (1999) | 0.002 | 0.967 | - | - |  |  |
| Grip strength (2002-5) | - | - | -0.058 | 0.364 |  |  |
| Lean leg mass (right) (1999) |  |  |  |  | 0.024 | 0.646 |

Adult ability - (NART result); Physical activity – average physical activity reported by questionnaire. Missing values imputed using multiple imputation

References

1. Carlin, J.B., et al., *Regression models for twin studies: a critical review.* Int J Epidemiol, 2005. **34**(5): p. 1089-99.
